# Use of high‐density SNP data to identify patterns of diversity and signatures of selection in broiler chickens

**DOI:** 10.1111/jbg.12228

**Published:** 2016-06-28

**Authors:** J.J. Stainton, B. Charlesworth, C.S. Haley, A. Kranis, K. Watson, P. Wiener

**Affiliations:** ^1^ The Roslin Institute and R(D)SVSUniversity of EdinburghEdinburghUK; ^2^ Institute of Evolutionary BiologySchool of Biological SciencesUniversity of EdinburghEdinburghUK; ^3^ MRC Human Genetics UnitMRC IGMMUniversity of EdinburghEdinburghUK; ^4^ Aviagen LtdEdinburghUK; ^5^ Present address: Scotland's Rural CollegeEdinburghUK

**Keywords:** Axiom chicken genotyping array, hitchhiking, poultry, regression method, selection mapping

## Abstract

The development of broiler chickens over the last 70 years has been accompanied by large phenotypic changes, so that the resulting genomic signatures of selection should be detectable by current statistical techniques with sufficiently dense genetic markers. Using two approaches, this study analysed high‐density SNP data from a broiler chicken line to detect low‐diversity genomic regions characteristic of past selection. Seven regions with zero diversity were identified across the genome. Most of these were very small and did not contain many genes. In addition, fifteen regions were identified with diversity increasing asymptotically from a low level. These regions were larger and thus generally included more genes. Several candidate genes for broiler traits were found within these ‘regression regions’, including *IGF1*,*GPD2* and *MTNR1AI*. The results suggest that the identification of zero‐diversity regions is too restrictive for characterizing regions under selection, but that regions showing patterns of diversity along the chromosome that are consistent with selective sweeps contain a number of genes that are functional candidates for involvement in broiler development. Many regions identified in this study overlap or are close to regions identified in layer chicken populations, possibly due to their shared precommercialization history or to shared selection pressures between broilers and layers.

## Introduction

In the absence of other factors, positive selection will eventually cause a beneficial allele to become fixed in a population, leading to a reduction in diversity in the population at the selected site. This hitchhiking effect (Maynard Smith & Haigh 1974) causes a statistical association between a selected site and neutral sites linked to it, with a pattern of reduced genetic variation around the selected site. Regions with low levels of variation may therefore indicate the action of positive selection.

Such regions that are candidates for targets of selection can be identified in a number of ways. Several methods estimate genome‐wide SNP diversities in a population and can be used to search for regions of the genome that have zero or very low diversity. The most straightforward approach is to directly measure genetic diversity across the genome. For example, Rubin *et al*. (2010) suggested a pooled diversity approach in which diversity in sliding windows across the genome is quantified using DNA pooled across populations/breeds. Low‐value outliers in the genomic distribution of diversity are considered to be candidates for selected positions. Another class of tests takes advantage of the linkage disequilibrium created by the hitchhiking effect, such as the long‐range haplotype (LRH) test (Sabeti *et al*. 2002) and the integrated haplotype score (iHS) test (Voight *et al*. 2006). Both of these tests utilize the extended haplotype homozygosity (EHH) statistic, which involves the probability that two chromosomes that carry the core haplotype are identical by descent (Sabeti *et al*. 2002). Another class of tests identifies regions with patterns of extreme allele frequencies or significantly distorted site frequency spectra (Kim & Stephan 2002; Nielsen *et al*. 2005; Pavlidis *et al*. 2010). The haplotype‐based procedures have greater power to detect ongoing selection (with intermediate frequencies of the favoured allele), while those based on allele frequency patterns have the greatest power to detect the recent fixation of alleles at sites linked to those under study (Qanbari *et al*. 2014). An alternative method takes advantage of the diversity pattern as a function of the position in the genome. As the distance from the selected site increases, the diversity levels in the population should increase. A test for this effect fits a regression to the diversity data to test the fit of the data to the theoretical expectation for a hitchhiking event (Wiener & Pong‐Wong 2011).

The identification of low‐diversity regions as signatures of selection has been previously applied to livestock using the above‐mentioned approaches. The greatest number of such studies has been performed in cattle, beginning in 2009 (see Gutiérrez‐Gil *et al*. 2015 for a review of 21 studies of European *Bos taurus*). The most recent studies have used very high‐density SNP chips and/or whole‐genome sequence data and have identified regions encompassing a number of genes associated with coat colour and other morphological features, as well as genes possibly associated with production traits (Ramey *et al*. 2013; Qanbari *et al*. 2014). Genomic scans for low‐diversity regions have also been performed in pigs (e.g. Rubin *et al*. 2012) and sheep (e.g. Gutiérrez‐Gil *et al*. 2014).

Modern chickens were domesticated before 6000 B.C., but within the last 70 years they have been split into separate lines specialized for egg production (layers) and meat production (broilers) (Muir *et al*. 2008). Both types are selected for a wide variety of traits, with layers focused on reproduction and broilers on meat yield and quality. This split was followed by large phenotypic changes; where these changes were associated with the appreciable effects of individual loci, the resulting genomic signatures of selection should be detectable by statistical techniques.

Several studies have scanned chicken genomes for regions of low diversity. Three of these (Rubin *et al*. 2010; Elferink *et al*. 2012; Qanbari *et al*. 2012) employed pooled diversity approaches. A region of approximately 40 kb was found to be nearly fixed for SNPs at the thyroid stimulating hormone receptor gene (*TSHR*) in commercial (broiler and white‐ and brown‐egg layer) and non‐commercial chicken breeds (Rubin *et al*. 2010). Elferink *et al*. (2012) used a similar approach, again across a range of broiler and white‐ and brown‐egg layer breeds, to identify regions showing strong evidence of selection. Qanbari *et al*. (2012) used a related statistical method to identify regions of low diversity in a brown‐egg layer line. Using a range of statistical approaches and data from a 60 K SNP chip (Zhang *et al*. 2012a,b) identified regions of low diversity in two lines of broilers under divergent selection for abdominal fat content, with the aim of identifying regions associated with this trait. Selection mapping approaches based on genetic differentiation between lines have also been applied in chickens (Gholami *et al*. 2014; in brown‐ and white‐egg layers; Stainton *et al*. 2015, in broilers).

The lower linkage disequilibrium in broilers than layers (particularly white‐egg layers) (Qanbari *et al*. 2010) requires a high‐density SNP panel to localize genomic signals. In this study, we examined genetic diversity in a broiler chicken line (Andreescu *et al*. 2007), which was genotyped using a SNP chip with ~581 K markers. These high‐density SNP data should allow the detection of regions showing evidence of selection during the recent development of broiler lines, with the lower levels of LD providing the potential to identify selective sweep regions with greater precision than would be possible using similar approaches in layer lines of chickens.

## Materials and methods

### Animals

In this study, we used birds from a broiler chicken line provided by Aviagen, a company primarily involved in broiler breeding. It is a closed dam line, selected with an inclusive breeding objective that balances reproductive and broiler performance, efficiency, welfare and health. The effective population size has been estimated as 50–200 (Andreescu *et al*. 2007). The broiler line was referred to as line 3 in previous studies (Andreescu *et al*. 2007; Stainton *et al*. 2015).

### Data

The SNP data set included 1513 male and female birds genotyped on the Affymetrix Axiom chicken genotyping array with a total of 580 954 SNPs (Kranis *et al*. 2013). Of these SNPs, 553 793 had known chromosome locations and were distributed across the autosomes and the Z chromosome. These SNPs were developed by sequencing 243 individual birds from 24 lines, including broilers, white‐egg layers, brown‐egg layers, inbred lines and an unselected layer line (Kranis *et al*. 2013). The distribution of SNPs per chromosome ranged from 603 to 102 502, with chromosome 16 containing the fewest SNPs. The data are hereafter referred to as the ‘600K’ data set.

### Quality control

Quality control protocols removed closely related individuals, including full sibs, half sibs and parents. The first individual family member found in the pedigree file was retained for analysis. Individuals with more than 10% of SNPs with no calls were also removed. SNPs not called in more than 10% of individuals were removed. Finally, SNPs on the Z chromosome were removed from the analysis. This left 264 individual birds and 530 247 SNPs for analysis.

### Statistical analysis

To investigate areas of low diversity, Nei's unbiased estimator of nucleotide site diversity (*h*) (Nei 1987) was calculated for each SNP using the following formula, where the sum is over the two alleles at a variable site:

$$h=\frac{2n(1-\sum_{i=1}^{2}X_{i}^{2})}{\left(2n-1\right)}$$

where

$$X_{i}=X_{\mathrm{ii}}+X_{\mathrm{ij}}/2$$

n = sample size, and *X*_ij_ = frequency of the (unordered) genotype *A*_i_ *A*_j_ in the sample (*i, j *=* *1,2, *i≠j*).

### Sliding windows

To reduce stochastic effects, the diversity results were averaged into overlapping sliding windows (Weir *et al*. 2005). A fixed window size method was used, which keeps a constant window size in terms of numbers of base pairs but with a varying number of SNPs per window (Stainton *et al*. 2015). A window size of 20 kbp was used, to achieve an average of approximately 10 SNPs per window (Table S1), and the central positions of adjacent windows were spaced 2 kbp apart. Any windows with fewer than two SNPs were removed from the analysis to prevent individual SNPs from biasing the results; this generated a total of 453 638 overlapping sliding windows. It is possible that some windows could be identical to one or more of the previous windows, if no SNPs were lost from the window and no new SNPs were added as the window moved along the genome. ‘Unique windows’ therefore refer to windows that were non‐identical in their SNP content with any other window.

### Regions of low diversity

To identify regions under selection, we first investigated windows with low diversity. ‘Regions’ were defined by identification of windows with zero or low (<0.005) diversity (‘zero‐diversity’ or ‘low‐diversity’ windows, respectively) that were physically adjacent to each other. A region could include a small gap of up to two windows to allow for windows that were discarded because they contained fewer than two SNPs. Even with such gaps, there were overlaps between some nearby windows, due to the markers shared between adjacent windows in an overlapping sliding window approach. We also required regions to be spread over more than one unique window, to reduce spurious signals.

### Proportion of zero‐diversity regions found for each type of region

When a fixed size is used to define windows, the number of SNPs in a region may vary dramatically across the genome. Additionally, the number of individual windows included in a region may vary, which will result in regions of different sizes. Smaller regions with fewer SNPs are more likely to include low‐diversity areas by chance, as these regions are less likely to contain at least one SNP with a small amount of variation. Therefore, it is useful to determine how many zero‐diversity regions have occurred in regions of a certain size and SNP count to assess how rare the identified regions are in the genome. To investigate this, the region size and SNP count were recorded for each zero‐diversity (target) region. For a given zero‐diversity region, all regions across the genome with that target size across the genome were then examined. The regions with SNP counts equal to that of the target region were extracted, and the total number of zero‐diversity regions within that set was counted. If there were fewer than 100 regions with a given target size and SNP count in the data set, the selection criterion was relaxed slightly (e.g. if the target region included 10 SNPs, diversity was assessed for all regions of the target size with 9–11 SNPs).

### Regression test and comparison with diversity results

A regression‐based analysis was performed in which asymptotic relationships between diversity and distance from a test position were estimated across the genome using the approach of Wiener & Pong‐Wong (2011). This method detects regions with patterns of variation consistent with positive selection: an asymptotic increase in variability *y* (measured as heterozygosity) with increasing distance (*x*) from a selected locus is modelled as *y *= *A *+ *B R* ^x^ (where *R* is the asymptotic rate of increase; *B* is the difference between heterozygosity at the test position and the asymptotic level; *A* is the asymptotic level of heterozygosity). Positive and increasing regressions (0 < *R *<* *1, *B *<* *0) were considered to be in the direction expected for positive selection. For the current implementation, the test position was moved in steps of 50 Kb across each chromosome and all markers within a fixed distance from this position (referred to as ‘bracket size’: 1, 5, and 10 Mb) were included in the asymptotic regression curve fitting (Gutiérrez‐Gil *et al*. 2014). A −log(p) value was determined for each test position, where p is the significance level associated with the asymptotic regression, as determined by the FITCURVE option in Genstat (Payne *et al*. 2009). The top 1% of −log(p) values for the respective bracket size were identified as potentially selected regions (‘regression regions’).

To reduce the stringency of the zero‐diversity criterion, all windows with diversity values <0.005 (‘low diversity’) were extracted and combined into regions by the methods described above for zero‐diversity regions and then evaluated for their correspondence (within 1 Mb distances) with regression regions.

### QTLs and genes within low‐diversity regions

We investigated the low‐diversity regions for QTLs and genes that were previously studied using two resources. For previously identified QTLs, we probed the Animal QTL Database (Hu *et al*. 2013). The database includes a total of 3919 QTLs in chickens, representing 297 traits, from 192 publications. The base pair positions reported in this database are on the Galgal4 build of the chicken genome (http://www.ensembl.org/Gallus_gallus/Info/Index). The chicken QTL database contains QTLs with large spans. Span is a map property defined by the animal QTL database, which represents a genomic area but does not incorporate any statistical confidence. To narrow down the possible QTLs for each low‐diversity region, we assigned the mid‐point of each QTL's span as the peak position. We searched the database and included any QTLs whose assigned peak position was located within a region. We used the following method for defining a ‘broiler QTL’: (i) they were assigned as ‘production’ QTLs in the Chicken QTL DB and (ii) they were directly related to meat production rather than egg laying. For example, growth, body weight, feed conversion ratio and breast muscle weight were all considered to be broiler QTL.

Ensembl Biomart (http://www.ensembl.org/biomart, accessed July 2015) was used to extract the genes in each region, with the *Gallus gallus* genes (Galgal4) data set. The numbers of genes present in the regions and their functions (if available) were recorded. An analysis of gene ontology (GO) term enrichment was performed using Gorilla (http://cbl-gorilla.cs.technion.ac.il/, accessed October 2015; Eden *et al*. 2009) for the genes in low‐diversity regions, using the options *Homo sapiens* (*Gallus gallus* was not available) and ‘Two unranked lists of genes’, where the ‘Target’ set of genes were protein‐coding genes with gene names identified in the low‐diversity regions and the ‘Background’ set of genes were all protein‐coding genes with gene names in the Galgal4 assembly, exported using Ensembl Biomart. Only genes with recognized names in the human genome assembly were considered in the analysis. Duplicate genes (e.g. due to different nomenclature) were also removed from the lists.

### Conversion of base pairs to centiMorgans

Base pair map locations were converted into centiMorgans to allow estimation of the amount of recombination occurring in each region, as the frequency of recombination between the selected site and neighbouring neutral variants is a critical parameter in determining the extent of reduction in variability caused by a selective sweep (Maynard Smith & Haigh 1974). Estimates of centiMorgan locations for each chromosome were obtained from Elferink *et al*. (2010). These locations were updated to the Galgal4 genome build using the marker ID in the *dbSNP* database (Sherry *et al*. 2001). Some centiMorgan locations did not have a nearby marker. These locations were estimated using the surrounding markers and the average recombination rate of the chromosome.

## Results

### Zero‐diversity regions

**Figure 1:**
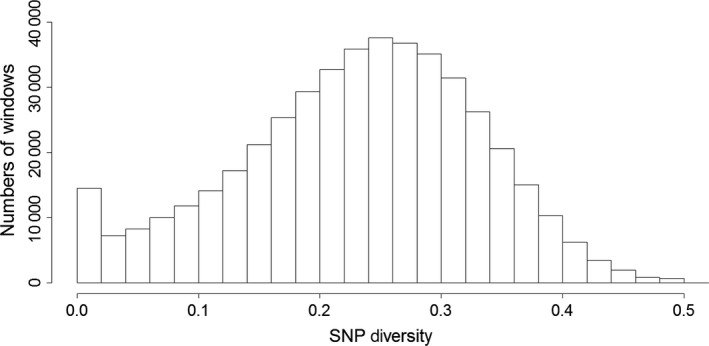
Distribution of diversity in 200 Kbp‐sized windows across the genome of broiler line 3, assessed using a ∼600 K SNP chip.

The use of regions of zero diversity in this data set is a very stringent criterion for detecting the signature of selection. To identify regions of very low diversity, the overall distribution of diversity in the data set was first investigated. This distribution is slightly positive‐skewed, with an excess of regions with a diversity less than 0.02 compared with a Gaussian distribution (Figure 1).

A total of seven regions (ZD.1–ZD.7) with zero diversity spread over two or more unique windows were found in the data set (Tables 1 and S2). These regions were present on chromosomes 1, 2, 4 and 5. The average zero‐diversity region was 0.031 Mb (SD = 0.009) in size. There were other zero‐diversity regions in the genome of similar size and SNP count to these seven regions (see column 14 of Table 1) but which did not meet the criteria defined above (i.e. at least two SNPs per 0.02 Mb window and regions made up of at least two unique windows). We used this larger set of regions to estimate the frequency of zero‐diversity regions in the genome for a given size and SNP count. For three of the seven zero‐diversity regions in Table 1, the proportion of genome‐wide regions of their size and SNP count that also had zero diversity was less than 0.4%. These included regions ZD.1 and ZD.7, which were the only regions with zero diversity found in regions of equivalent size and SNP count across the genome. For the other four regions, the number of zero‐diversity regions of equivalent size and SNP count ranged from 5 to 22. Some selected examples of the seven zero‐diversity regions are described below. This includes the largest region in terms of physical size, the region containing the most QTL peak positions, the most gene rich region and the region for which the proportion of zero‐diversity regions of equivalent size and SNP count was smallest.

**Table 1:** Zero‐diversity regions spread over more than one unique window present in the 600 K data set in line 3, showing numbers of SNPs, QTLs and genes

Region no	Chr	Start (Mb)	End (Mb)	Size (Mb)	Start (cM)	End (cM)	Size (cM)	SNPs	QTLs	Broiler QTLs	Genes	Zero‐diversity regions	Total regions	Proportion of zero diversity (%)
ZD.1	1	65.972	66.00	0.028	130.54	130.68	0.14	11	0	0	2	1	31 207	0.0032
ZD.2	1	77.584	77.608	0.024	154.75	154.78	0.03	3	0	0	1	19	870	2.18
ZD.3	1	139.28	139.328	0.048	276.04	276.05	0.01	4	3	2	0	9	329	2.74
ZD.4	2	52.782	52.808	0.026	124.72	124.72	0.00	3	0	0	1	18	693	2.60
ZD.5	2	135.362	135.386	0.024	267.83	267.87	0.04	4	0	0	1	5	1440	0.35
ZD.6	4	85.26	85.298	0.038	175.90	176.02	0.12	3	0	0	7	22	352	6.25
ZD.7	5	48.906	48.936	0.03	108.76	108.79	0.03	7	0	0	0	1	2747	0.04

The location includes estimates for both Mb and cM. The final three columns are the number of zero‐diversity regions of this size and SNP count, the total number of regions of this size and SNP count, and the proportion of regions of this size and SNP count with zero diversity.

#### Region ZD.1 (1: 65.97–66.00 Mb) – Region with the lowest proportion of zero‐diversity regions of equivalent size and SNP count

**Figure 2:**
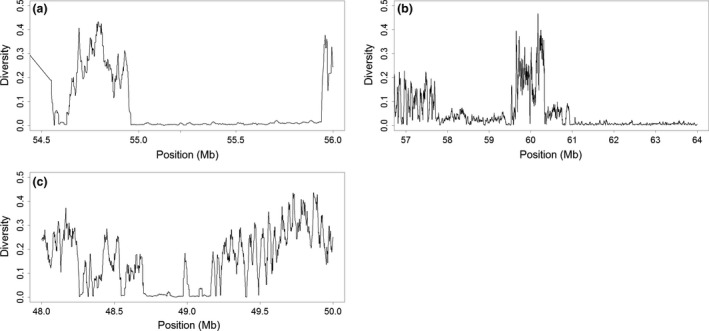
Diversity across putatively selected regions, with significant asymptotic regressions. (a) Diversity values in region Reg.2 on chromosome 1 (diversity values less than 0.005 between 54.5 and 56 Mb). (b) Diversity values in region Reg.6 on chromosome 4 (diversity <0.005 between 58 and 62 Mb). (c) Diversity values in region Reg.7 on chromosome 5 (diversity <0.005 between 48 and 49 Mb).

Of the 31 207 regions of equivalent size and SNP number, this 0.028 Mb (0.14 cM) region on chromosome 1 was the only one with zero diversity (Figure 2a). While region ZD.7 was also unique, the proportion of zero‐diversity windows with an equivalent size and SNP number to ZD.1 is extremely low (0.0032%), so a chance finding of a zero‐diversity region of this size and SNP count is particularly unlikely. Additionally, this is the largest region in terms of centiMorgans, on a chromosome with a relatively low recombination rate (2.1 cM/Mb, compared with the per‐chromosome average of 5.1 cM/Mb) (Elferink *et al*. 2010). No QTL peak positions or coding genes were found in this region, although two microRNAs with unknown function are located there.

#### Region ZD.3 (1: 139.28–139.33 Mb) – Largest region in terms of physical size and region with greatest number of QTL peak positions

A 0.048 Mb (0.01 cM) region of zero diversity on chromosome 1 was the largest region in terms of physical size. No genes were found in this region. However, this region contains the most QTL peak positions (3) of all the zero‐diversity regions. Two of these can be classed as broiler QTL and are associated with body weight and breast muscle weight traits. The proportion of regions of equivalent size and SNP count with zero diversity was 2.74%.

#### Region ZD.6 (4: 85.26–85.30 Mb) – Region containing the most genes

A 0.038 Mb (0.12 cM) region of zero diversity found on chromosome 4 (Figure 2b) contains seven genes. No QTLs were found with a peak position in this region. A total of 23 QTLs had spans overlapping the region, nine of which could be classed as broiler traits. These include feed conversion ratio, body weight, abdominal fat weight and thigh muscle weight. The proportion of regions of equivalent size and SNP count with zero diversity was 6.25%.

### Low‐diversity regions

Regions identified using the diversity <0.005 threshold for windows included 321 genes (297 protein coding, Table S3) and 59 QTL for broiler traits (Table S4). The GO enrichment analysis of annotated protein‐coding genes was based on 281 target genes and 11 674 background genes, including those for which gene names were recognized by Gorilla and that had associated GO terms. The genes in these regions were enriched (raw p < 1E‐3) for three GO terms, although none survived multiple testing correction due to the large number of background genes tested. The ‘process’ term ‘muscle cell development’ was enriched by 4.77‐fold (p = 6.24e‐4), and the term ‘regulation of protein oligomerization’ was enriched by 8.75‐fold (p = 9.57e‐4). The ‘component’ term ‘plasma membrane part’ was enriched by 1.50‐fold (p = 5.22e‐4). The genes that were associated with muscle cell development include *RAMP2*,* SGCZ*,* SGCD*,* BVES*,* NFATC3*,* IGF1* and *FHL2* and those that were associated with regulation of protein oligomerization include *IDE*,* IGJ*,* SRC* and *BCL11A*.

### Regression analysis

Twenty‐one regions were identified by the regression method from one or more bracket sizes, across 14 chromosomes (bracket size = 1 Mb: 16 regions, bracket size = 5 Mb: four regions, bracket size = 10 Mb: two regions). One region overlapped a zero‐diversity region (T.7), while the remainder were not within 1 Mb of a zero‐diversity region. Fifteen of these regions (Reg.1–Reg.15) were, however, located within 1 Mb of a low‐diversity region (diversity <0.005) (Tables 2 and S5). The average size of these 15 regions was 0.87 Mb, substantially greater than that for the zero‐diversity regions.

**Table 2:** Regions displaying diversities less than 0.005 and that are located within 1 Mb of a region identified by the regression approach (top 1% of −log(p) values), over one or more bracket sizes (1 Mb, 5 Mb and 10 Mb)

Region no	Chr	Start (Mb)	End (Mb)	Size (Mb)	Start (cM)	End (cM)	Size (cM)	Bracket size (Mb)	QTL	Broiler QTL	Genes
Reg.1	1	4.526	4.806	0.280	7.58	7.80	0.22	5	0	0	0
Reg.2	1	54.554	55.670	1.116	102.55	105.29	2.75	1	2	2	22
Reg.3	1	182.492	182.872	0.380	347.10	347.31	0.21	5	0	0	2
Reg.4	1	184.152	184.174	0.022	348.13	348.19	0.06	5	0	0	2
Reg.5	2	26.906	27.314	0.408	70.54	70.66	0.12	1	0	0	2
Reg.6	4	58.526	62.148	3.622	127.14	132.53	5.40	10	6	4	40
Reg.7	5	48.250	49.418	1.168	108.04	109.76	1.73	1	5	3	15
Reg.8	5	50.926	50.954	0.028	115.44	115.50	0.05	1	0	0	0
Reg.9	5	55.928	56.136	0.208	141.63	142.65	1.02	1	1	1	5
Reg.10	7	35.378	36.214	0.836	101.97	103.00	1.03	1	1	1	17
Reg.11	11	1.464	4.022	2.558	3.36	6.98	3.62	5, 10	4	2	70
Reg.12	15	3.236	3.568	0.332	3.63	4.05	0.41	1, 5	1	1	4
Reg.13	20	5.424	5.662	0.238	21.00	22.48	1.48	1	0	0	18
Reg.14	25	0.906	1.762	0.856	36.25	56.22	19.97	1	0	0	84
Reg.15	28	3.778	4.716	0.938	52.01	54.00	1.99	1	1	1	42

## Discussion

Domesticated chickens, specialized for their role in either egg or meat production, have been produced by artificial selection, which has caused large phenotypic and genotypic changes. In this study, we have identified putative signatures of selection in broiler chickens by analysing genetic diversity patterns in a high‐density data set of genome‐wide SNPs. Seven zero‐diversity regions were found in this data set. Furthermore, fifteen low‐diversity regions (one of which was also a zero‐diversity region) were found within 1 Mb of regions in which diversity increased asymptotically away from a central position with low diversity, consistent with the action of a selective sweep.

### Region identification method

The fixed‐size window method we employed and the method of determining regions mean that the regions identified can vary in both size and SNP count. Certain size and SNP count combinations are more likely to include regions of zero diversity. For example, a small region with only three SNPs will be more likely to have diversity of zero than a large region with 10 SNPs. To deal with this issue, we calculated how many regions of the same size and SNP count had zero diversity. Two regions (ZD.1 and ZD.7) were the only zero‐diversity regions with their respective size and SNP count; ZD.1 was particularly unusual as it was the only zero‐diversity region among 31 207 regions of the same size and SNP count.

### Characterization of regions showing reduced diversity

Several of the regression regions contained candidate genes that may be under selection in broilers. Region Reg.2 on chromosome 1 contains the *insulin‐like growth factor 1 (IGF1)* gene*,* which has been shown to be associated with growth, skeletal integrity and feeding traits in chickens (Amills *et al*. 2003; Zhou *et al*. 2005). Region Reg.10 on chromosome 7 contains the *glycerol‐3‐phosphate dehydrogenase 2* (*GPD2*) gene. This gene is associated with increased glycerol production, leading to an accumulation of fat, and was found in a previous study to be expressed at higher levels in chickens selected for low abdominal fat content (Resnyk *et al*. 2013). However, there were no obvious candidate genes in the zero‐diversity regions. These regions were substantially smaller than most of those identified using the regression approach and therefore contain fewer genes. The genes found in low‐diversity regions did, however, suggest enrichment for muscle cell development functions, consistent with the high muscle content of broiler chickens and the known changes in broiler carcass composition over the last 50+ years (Collins *et al*. 2014).

A number of the regions identified were located near regions showing evidence of selection in one or more previous studies of chickens (Rubin *et al*. 2010; Elferink *et al*. 2012; Zhang *et al*. 2012a,b) (see Table 3 legend for further details on comparison of regions). Region Reg.11 (chrom 11) is near regions of reduced diversity in several separate studies, including broilers and brown‐ and white‐egg layers. This relatively large region is very gene rich, overlapping 70 genes, and contains the peak positions for two QTL related to abdominal fat content. None of the genes in this region were obvious functional candidates for broiler traits although the *FTO* gene, associated with body weight and composition in chickens (Jia *et al*. 2012) and obesity in humans (Frayling *et al*. 2007), is located ~1 Mb downstream.

**Table 3:** Regions identified in previous studies that are located within 1 Mb of the zero‐diversity or regression regions identified in this study

Region no	Chr	Start (Mb)^a^	End (Mb)^a^	Size (Mb)	Previous studies^b^ that identified region within 1 Mb of current study (bold indicates direct overlap)
Reg.1	1	4.526	4.806	0.28	B (CH), G
Reg.2	1	54.554	55.670	1.116	**A (LR,CB)**,** B (BR,BRS)**,** C (fat)**,** E**, G
Reg.3	1	182.492	182.872	0.38	**B (BRD)**
Reg.4	1	184.152	184.174	0.022	B (CH), F
Reg.5	2	26.906	27.314	0.408	A (**CB**,AD), B(DM,CM,NCM,BR,LR,DU,BRS), **D (lean)**, G
Reg.6	4	58.526	62.148	3.622	A (AD,LR), **B (DM,CM,NCM,BR,LR,BRS,BRD,DNB)**, E, **F**,** G**
Reg.7	5	48.250	49.418	1.168	A (AD), B (DU), **F**
Reg.8	5	50.926	50.954	0.028	
Reg.9	5	55.928	56.136	0.208	D (fat line), **G**
Reg.10	7	35.378	36.214	0.836	**A (AD,LR)**,** B(DM,CM,NCM,BR,LR,BRS,BRD)**, D (fat), F
Reg.11	11	1.464	4.022	2.558	**A (CB,AD,LR)**, B(**DM,CM,NCM,BR,LR,DU**,CH,**BRS,BRD,BL,DPB**), **C (lean)**, F, G
Reg.12	15	3.236	3.568	0.332	A (LR), C, F, G
Reg.13	20	5.424	5.662	0.238	A (CB), B (BR,BRD), F, G
Reg.14	25	0.906	1.762	0.856	B (**DM,CM,NCM**,CH), **F**
Reg.15	28	3.778	4.716	0.938	**A (CB,LR)**,** B (BR,BRS)**, E
ZD.1	1	65.972	66.00	0.028	A (LR), D (fat), F
ZD.2	1	77.584	77.608	0.024	B (CH)
ZD.3	1	139.28	139.328	0.048	B (CH)
ZD.4	2	52.782	52.808	0.026	A (**AD**,CB), B (CM, BR, BRS, BRD)
ZD.5	2	135.362	135.386	0.024	E
ZD.6	4	85.26	85.298	0.038	G
ZD.7	5	48.906	48.936	0.03	B (DU), F

a For studies A–E (see listing below), positions were transformed using liftOver (https://genome.ucsc.edu/cgi-bin/hgLiftOver) from galGal4 to the previous chicken genome assembly (galGal3) for comparison; for studies F and G (see listing below), galGal4 positions were compared directly. Regarding the liftOver procedure, the Start and End positions were separately converted to the previous assembly. In a few cases, either the Start or End position could not be identified in which case it was estimated based on the size of the region in the new assembly (in cases where both Start and End positions were successfully converted, region sizes were very similar between the two assemblies).

b Previous studies (table numbers below refer to the original publications):

(A) Study of commercial broilers and layers and non‐commercial chickens (Rubin *et al*. 2010). Includes regions showing ZH_P_ < −4 (Table S3). AD = all domestic; CB = commercial broilers; LR = layers (including both white and brown egg).

(B) Study of commercial broilers and layers and non‐commercial chickens (Elferink *et al*. 2012). Includes regions showing ZH_P_ < −4 (Table S12). DM = domesticated; CM = commercial; NCM = non‐commercial; BR = broiler; LR = layer; DU = Dutch; CH = Chinese; BRS = broiler sire; BRD = broiler dam; WL = white layer; BL = brown layer; DCF = Dutch country fowls; DPB = Dutch polish & bearded; DNB = Dutch new breeds.

(C) Study of two lines of broilers divergently selected for abdominal fat content (Zhang *et al*. 2012a). Includes regions identified showing extreme low heterozygosity or differences in allele frequency between ‘fat’ and ‘lean’ lines (Table 3). fat = line selected for increased abdominal fat levels; lean = line selected for decreased abdominal fat.

(D) Study of two lines of broilers divergently selected for abdominal fat content (Zhang *et al*. 2012b). Includes regions showing evidence of selection based on the REHH test (Table 3). fat = line selected for increased abdominal fat levels; lean = line selected for decreased abdominal fat.

(E) Study of a brown commercial layer line (Qanbari *et al*. 2012). Includes regions showing extreme low heterozygosity (Table S1).

(F) Study of commercial layers and non‐commercial breeds (Gholami *et al*. 2014). Includes regions in upper 1% F_ST_ distribution in comparison of brown layers and white layers (Table S4).

(G) Study of commercial broiler lines (Stainton *et al*. 2015). Includes regions showing statistically significant between‐line differentiation (Table 3).

Another regression region, Reg.6 (chrom 4; the largest identified in this study), is also near regions showing evidence of selection in several previous studies, again including broilers and brown‐ and white‐egg layers (Table 3). This region also overlaps with a highly differentiated region identified in our previous study of nine broiler lines (Stainton *et al*. 2015) (see below). Forty genes overlap with this region as well as four broiler QTL related to feed intake, shank length and meat quality. One of these genes is melatonin receptor 1A, *MTNR1A*. Melatonin has previously been associated with body weight and abdominal fat reduction in rats (Wolden‐Hanson *et al*. 2000) by regulating brown adipose tissue metabolism (Tan *et al*. 2011). Melatonin has also been associated with reproductive traits in chickens, including skeletal development in the embryos of laying hens (Taylor *et al*. 2013), number of eggs and age at first egg (Li *et al*. 2013).

Six of the seven zero‐diversity regions are within 1 Mb of a region identified in previous selection mapping studies, but only one (ZD.4, chrom 2) overlaps directly (Table 3). This region is found near regions of low diversity identified primarily in broilers in two studies (Rubin *et al*. 2010; Elferink *et al*. 2012); however, there were no obvious functional candidate genes found in or close to this region. There are no QTLs or coding genes that overlap ZD.1 (chrom 1), the region that is the only zero‐diversity region of its size and SNP count of a total of 31 207 regions, although two microRNAs are found there.

The proximity of the regions identified in this study to those identified in previous studies of chickens, including both broilers and layers, could be due to their shared pre‐ or postcommercialization histories or to shared recent selection pressures between commercial lines. More detailed comparisons of individual lines are necessary to determine whether particular haplotypes are shared across lines in these regions, which would support a common history.

In our previous study of nine broiler chicken lines (including line 3, analysed in the current study) genotyped using a low‐density SNP chip, a number of highly differentiated regions were identified in multiple lines (Stainton *et al*. 2015). Eight of the 15 regression regions were located within 1 Mb of the highly differentiated regions (Table 3), consistent with the theoretical expectation of negative relationship between diversity and *F*_ST_ (Charlesworth 1998; Cruickshank & Hahn 2014). Two of the regression regions directly overlapped the differentiated regions: Reg.6 (chrom 4), which overlapped differentiated regions 24 and 25, and region Reg.9 (chrom 5), which overlapped differentiated region 29 (Table 3 from Stainton *et al*. 2015). One of the zero‐diversity regions (ZD.6) was located within 1 Mb of a differentiated region (region 27), but they did not directly overlap. These results suggest that much of the selection we detected in the current study is related to differentiation between different broiler lines.

## Conclusions

A number of putative selection signatures were identified in a broiler chicken line genotyped with high‐density SNPs. Several small regions with zero‐diversity were identified, while a larger number of regions were identified using a regression approach and using a less stringent diversity threshold. Our results suggest that the identification of zero‐diversity regions is too restrictive for characterizing regions under selection and that the use of the asymptotic regression method is more promising. The use of a SNP array probably contributed to the limitations of the zero‐diversity approach because such arrays may be enriched for variants that are segregating only in certain populations/breeds, and may thus overestimate variation, particularly in low‐diversity regions. Some of the regions we identified overlapped with QTLs related to broiler traits, and nearly all were found near selection signatures found by previous studies. A few of the identified regions contained candidate genes: one of the regression regions contains the *IGF1* gene, known to be involved in growth and a likely candidate gene for broiler selection, while another overlaps *MTNR1A*, which has been associated with body composition and reproductive traits. There was also a suggestion of enrichment for genes related to muscle development in the low‐diversity regions. Further studies should examine the genomic diversity of commercial chickens and phenotypic associations for the identified regions.

## Supporting information


**Table S1** Characteristics of broiler line 3 genome assessed by a ∼ 600 K SNP chip, for different window sizes.

**Table S2** Genes extracted from zero diversity regions (Table 1).

**Table S3** Genes extracted from low diversity (<0.005) regions.

**Table S4** QTL extracted from low diversity regions.

**Table S5** Genes extracted from low diversity regions that overlap with regions identified using regression approach (Table 2).
