# Supplementary material for: Use of high‐density SNP data to identify patterns of diversity and signatures of selection in broiler chickens
Source: J Anim Breed Genet. 2016 Jun 28;134(2):87–97. doi: 10.1111/jbg.12228 (PMC5363361; doi:10.1111/jbg.12228)
Supplement: Supplementary file 1 — Table S1 Characteristics of broiler line 3 genome assessed by a ∼ 600 K SNP chip, for different window sizes. [file JBG-134-87-s001.docx]

Supplementary table 1: Characteristics of broiler line 3 genome assessed by a 600K SNP chip, for different window sizes.

| **Window Size** | **Increment** | **Number of Windows** | **Zero diversity Windows** | **SNPs per Window** | | |
| --- | --- | --- | --- | --- | --- | --- |
|  |  |  |  | **mean** | **s.d.** | **range** |
| 10000 | 1000 | 897057 | 1321 | 5.898664 | 2.82436 | 2 – 44 |
| 20000 | 2000 | 455370 | 71 | 11.640500 | 5.02641 | 2 - 78 |
| 30000 | 3000 | 304320 | 39 | 17.416400 | 7.22441 | 2- 118 |
| 100000 | 10000 | 91753 | 16 | 57.687480 | 22.27209 | 2- 312 |
| 200000 | 20000 | 45823 | 7 | 115.20340 | 42.85156 | 2- 525 |
| 300000 | 30000 | 30463 | 0 | 172.77000 | 62.7676 | 5 – 772 |
| 840000 | 85000 | 10570 | 0 | 482.55250 | 164.277 | 140 - 1971 |
